# Design of a continuous quality improvement program to prevent falls among community-dwelling older adults in an integrated healthcare system

**DOI:** 10.1186/1472-6963-9-206

**Published:** 2009-11-16

**Authors:** David A Ganz, Elizabeth M Yano, Debra Saliba, Paul G Shekelle

**Affiliations:** 1 VA Greater Los Angeles HSR&D Center of Excellence, 16111 Plummer Street, Sepulveda, CA 91343, USA; 2 Geriatric Research, Education and Clinical Center, Veterans Affairs Greater Los Angeles Healthcare System, 11301 Wilshire Boulevard, Los Angeles, CA 90073, USA; 3 David Geffen School of Medicine at the University of California at Los Angeles, 10833 Le Conte Avenue, Los Angeles, CA 90095, USA; 4 School of Public Health, University of California at Los Angeles, 650 Charles E. Young Drive South, Los Angeles, CA 90095, USA; 5 Borun Center for Gerontological Research, University of California at Los Angeles and Los Angeles Jewish Home, 10945 Le Conte Avenue, Suite 2339, Los Angeles, CA 90095, USA

## Abstract

**Background:**

Implementing quality improvement programs that require behavior change on the part of health care professionals and patients has proven difficult in routine care. Significant randomized trial evidence supports creating fall prevention programs for community-dwelling older adults, but adoption in routine care has been limited. Nationally-collected data indicated that our local facility could improve its performance on fall prevention in community-dwelling older people. We sought to develop a sustainable local fall prevention program, using theory to guide program development.

**Methods:**

We planned program development to include important stakeholders within our organization. The theory-derived plan consisted of 1) an initial leadership meeting to agree on whether creating a fall prevention program was a priority for the organization, 2) focus groups with patients and health care professionals to develop ideas for the program, 3) monthly workgroup meetings with representatives from key departments to develop a blueprint for the program, 4) a second leadership meeting to confirm that the blueprint developed by the workgroup was satisfactory, and also to solicit feedback on ideas for program refinement.

**Results:**

The leadership and workgroup meetings occurred as planned and led to the development of a functional program. The focus groups did not occur as planned, mainly due to the complexity of obtaining research approval for focus groups. The fall prevention program uses an existing telephonic nurse advice line to 1) place outgoing calls to patients at high fall risk, 2) assess these patients' risk factors for falls, and 3) triage these patients to the appropriate services. The workgroup continues to meet monthly to monitor the progress of the program and improve it.

**Conclusion:**

A theory-driven program development process has resulted in the successful initial implementation of a fall prevention program.

## Background

Falls are common in older people, occurring annually in one quarter to one third of community-dwelling adults age ≥ 65 [1]. These falls pose a serious problem, both because of associated injuries (e.g., hip fractures) [2] and because of the falls' psychological impact on patients [3]. Older adults may restrict their activities in response to a fall, leading to a loss of independence and ability to carry out life's routine tasks. Research evidence has shown that both gentle exercise to improve strength and balance and multifactorial fall prevention programs can reduce future falls in community-dwelling older adults who participate in randomized, controlled trials [4,5]. These fall prevention programs often involve a range of providers and interventions to address the various contributing risks that lead to a fall [6]. Risk factors uncovered during an evaluation lead to targeted interventions (such as exercise for lower extremity strength and improved balance, discontinuation of medications that increase fall risk, or installation of grab bars in the bathroom).

Clinical practice guidelines recommend multifactorial fall prevention strategies for patients at high risk of subsequent falls [7]. However, recent attempts to implement fall prevention activities in routine practice have met with mixed results [8-11]. Even in situations where strong evidence supports a program, mixed results in implementation may relate to the context in which the program was implemented, or the process used to facilitate program implementation [12]. This article reports on the process we used to develop a fall prevention program for community-dwelling older adults served by our local healthcare system, as well as key contextual features of our system. We also lay out the theoretical support for our program development process. We hope to stimulate discussion regarding the merits of different strategies for implementing evidence into routine care, particularly for complex programs that require a confluence of support from diverse stakeholders.

## Methods

### Ethics Approvals

This study conforms to the ethical principles in the Helsinki Declaration, and received ethics approval from the VA Greater Los Angeles Healthcare System (PCC 2008-010128) and University of California at Los Angeles (G08-06-103-01) Institutional Review Boards. Because of the minimal risk nature of this project, the Institutional Review Boards waived the requirement for written informed consent.

### Setting

The Veterans Affairs Greater Los Angeles Healthcare System (VAGLAHS) is an integrated healthcare system serving veterans living in a catchment area that spans the metropolitan Los Angeles area. The system includes one acute care hospital with clinics, two community living centers, two ambulatory care centers offering primary care and some specialty services, and ten community-based outpatient clinics that offer mostly primary care. VAGLAHS serves a disproportionately older population; nearly one-quarter of all patients are age ≥ 75, and therefore, a large group of VAGLAHS patients are at risk for falls. In absolute terms, VAGLAHS served a total of 19,257 unique patients age ≥ 75 in its primary care clinics over a two-year period (September 1, 2005 to August 31, 2007) (unpublished data).

VAGLAHS is a globally budgeted, capitated healthcare system. Virtually all healthcare providers are salaried, and all clinicians use a shared electronic health record. VAGLAHS is one of five healthcare systems in the Desert Pacific Healthcare Network, which serves Southern California and Southern Nevada. This network, which finances VAGLAHS' operations, is one of 21 networks in the United States Veterans Health Administration (VA).

The national VA Office of Quality and Performance runs the External Peer Review Program (EPRP) to monitor the quality of care provided by VA facilities. In the case of fall prevention, quality indicators previously developed by the RAND Assessing Care of Vulnerable Elders (ACOVE) project (for ACOVE methods, see sources [13] and [14]) were adopted by the EPRP on a pilot basis for VA outpatients age ≥ 75. These quality indicators are called VA "supporting indicators" because they are not being used to judge facility or network performance, but rather to provide initial information to the Office of Quality and Performance regarding quality of care. Quality indicators are monitored by reviewing a random sample of medical records at VA facilities.

There are two supporting indicators for fall prevention: the first indicator states that outpatients age ≥ 75 should be asked about falls in the preceding year. To determine adherence to this indicator, medical record reviewers are asked: "Within the past twelve months, was the patient asked about the presence/absence of any falls within the preceding 12 months?" The second indicator states that a basic fall evaluation should be performed on outpatients age ≥ 75 with two falls, or at least one fall with injury requiring treatment, in the past year. To determine whether a basic fall evaluation was performed, reviewers search the medical record for documentation of five elements: 1) circumstances of the fall, 2) medications the patient is taking, 3) relevant chronic conditions, 4) diagnostic plans/therapeutic recommendations, and 5) documentation of action taken as appropriate. Although these quality indicators are monitored at the national level, they are not in the first tier of performance measures for which VA managers are held accountable. However, facility-level quality of care scores for these indicators are available internally to VA managers, clinicians, and researchers on the VA Intranet. In addition, through a data use agreement, researchers may obtain more detailed data from the Office of Quality and Performance.

**Figure 1:**
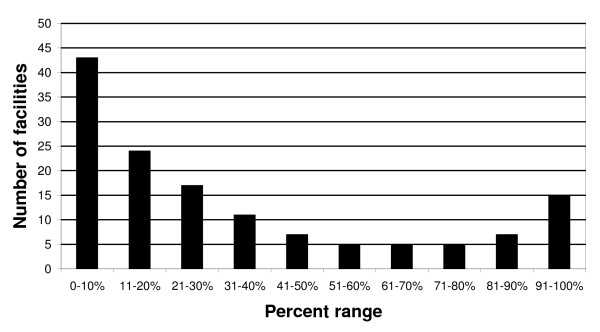
**Adherence of 139 VA facilities to asking outpatients age ≥ 75 about falls in the past year**. Data are derived from VA External Peer Review Program data from Fiscal Year 2007 (October 1, 2006 to September 30, 2007) based on a review of a random sample of medical records at each facility. The Y-axis indicates the number of facilities in a particular adherence range. The X-axis groups facilities by the percent of patients who were asked about falls.

We obtained data for the first quality indicator for falls - asking outpatients age ≥ 75 about falls in the past year -- for Fiscal Year 2007 (October 1, 2006 to September 30, 2007), the first year during which these data were collected. There was marked variation between VA facilities (such as VAGLAHS) in how often outpatients age ≥ 75 were asked about falls in the past year (Figure 1). On average, 33% of patients nationwide were asked about falls (facility N = 139, patient N = 27899), similar to a 25% rate seen among vulnerable older adults in two managed care organizations outside the VA system [15]. Although this quality indicator was not designed to compare facilities with one another, additional analyses (not shown) revealed that our local facility's performance needed to improve, regardless of whether the national average, or a higher benchmark, was set as the target.

The second quality indicator -- performing a basic fall evaluation on outpatients age ≥ 75 with two falls (or one fall with injury requiring treatment) in the past year - was satisfied in 36% of eligible cases nationally (Patient N = 899). The sample size for this indicator was too small to compare our local facility performance to the national average. However, this indicator is coupled to the previous one - asking more adults age ≥ 75 about falls in the preceding year (indicator #1) will result in the detection of more high-risk cases requiring a basic evaluation (indicator #2). Thus, improving the quality of care for falls at our facility requires both better detection of patients with a significant fall history and better ways to address fall risk factors among high-risk patients.

Table 1 summarizes the various contextual factors that might affect successful implementation of a fall prevention program at VAGLAHS. The categories in Table 1 come from a systematic review of factors critical to successful implementation of prevention programs, based on data from actual attempts at implementation [16].

**Table 1:** Factors potentially affecting implementation of the fall prevention program in the VA Greater Los Angeles Healthcare System (VAGLAHS) (factors adapted from Durlak and DuPre [16]).

Key factors	Status of these factors in fall prevention program
I. Community level factors	

A. Prevention theory and research	There is a well developed research literature on fall prevention. Theory, however, is less well developed.

B. Politics	The political environment has some awareness of falls [29], although in the United States this awareness does not rise to the level of other medical concerns such as cancer and heart disease.

C. Funding	There is some funding available for fall prevention activities from United States government organizations such as the Centers for Disease Control and Prevention as well as philanthropic organizations (e.g., the Archstone Foundation). However, funding for these activities does not parallel the availability of funds for other conditions.

D. Policy	Current policies by governmental organizations provide a small but clear level of support for fall prevention activities [29].

II. Provider characteristics	

A. Perceived need for innovation	Informal interviews with providers (physicians, nurses) in the ambulatory care setting as well as facility leadership suggest the perceived need for innovation at VAGLAHS is moderate to high.

B. Perceived benefits of innovation	Different individuals perceive different benefits from an enhanced fall prevention program. Some perceive the potential to save the facility money in reduced costs from inpatient hospital stays due to injuries. Others perceive the possibility of an improvement in quality of care due to better access to fall prevention services for patients. Yet others note that efficiency might be improved by streamlining the array of fall prevention services already in existence.

C. Self-efficacy	Providers' self-efficacy in preventing falls is unknown. Investigators at VAGLAHS are funded to develop a survey of primary care providers that will assess this issue.

D. Skill proficiency	Providers' skill proficiency in implementing fall prevention activities is unknown. Investigators at VAGLAHS are funded to develop a survey of primary care providers that will assess this issue.

III. Characteristics of the innovation	

A. Compatibility	The fall prevention program being developed is designed to be compatible with existing work processes at VAGLAHS. At least initially, it will not require hiring new individuals, changing existing technology, or reshaping work culture. Instead, it uses an already existing service within the organization in a novel way.

B. Adaptability	The program is fundamentally adaptable. The script that nurses read to patients as part of the telephonic assessment (see Additional File 2) may be modified. The places to which patients may be referred based on the telephonic evaluation may be altered. Multiple provider types (e.g., physicians, nurses, social workers) may place a request for a telephonic fall risk assessment, although in some cases, individuals may need physician or mid-level practitioner approval to place a request.

IV. Factors relevant to the prevention delivery system: organizational capacity	

A. General organizational factors	

1. Positive work climate	The work climate of VAGLAHS relative to other institutions is unclear. Attempts to measure the VA's work climate in a reproducible way are ongoing [30].

2. Organizational norms regarding change	VAGLAHS is open to small, incremental changes but probably less open to radical system redesign.

3. Integration of new programming	VAGLAHS has adequate organizational slack to integrate a new program on a pilot basis. The ability of VAGLAHS to implement a new program with high workload is doubtful.

4. Shared vision	The organization has a shared vision of providing improved services to veterans.

B. Specific practices and processes	

1. Shared decision-making	The interest in shared decision-making at VAGLAHS seems to be high. The two leadership meetings to discuss whether and how the fall prevention program should be developed and implemented were well-attended, including members of senior leadership.

2. Coordination with other agencies	Coordination between VAGLAHS and outside agencies that provide fall prevention activities (e.g., community senior centers) is difficult. The VA's electronic health record provides a strong incentive to coordinate activities internally. An exception to this relevant to fall prevention is the use of home care services (including home physical therapy and home safety evaluations), which can be ordered through the VA electronic health record but then may be contracted to a private home health agency, and financed either via a veteran's health insurance benefits or by direct payment from the VA to an outside agency.

3. Communication	Communication is facilitated by a common internal e-mail system and phone directory.

4. Formulation of tasks	Task formulation was enhanced by the Chief of Staff's chartering a workgroup composed of members from different disciplines relevant to fall prevention (ambulatory care, geriatrics, physical medicine & rehabilitation, nursing, performance improvement, research).

C. Specific staffing considerations	

1. Leadership	The Chief of Staff of VAGLAHS is a geriatrician and therefore has a heightened appreciation of the need for improved quality of care with respect to fall prevention. He is supportive of the intervention.

2. Program champion	The first author is acting as program champion. This champion status is protected for 5 years through salary support from a VA Career Development Award whose specific aims include the development and implementation of a fall prevention program at VAGLAHS. The first author's career development is supported by two mentors (PGS and DS) and an advisory committee (including EMY).

3. Managerial/supervisory/administrative support	Administrative support for the development of the fall prevention program is limited.

V. Factors related to the prevention support system	

A. Training	The first author has provided continuing medical education about fall prevention to VAGLAHS providers through lectures at various sites within the system. VAGLAHS has a variety of more general training programs that could be harnessed to increase awareness of the importance of fall prevention.

B. Technical assistance	Currently, the first author provides technical assistance to clinicians involved with the project on an ad hoc basis. Developing more formal technical assistance for providers will become necessary if the program advances beyond its pilot phase.

### Program development

In order to improve the quality of care for fall prevention provided to outpatient veterans at VAGLAHS, we envisioned a program development process that would harmonize stakeholder interests towards the goal of producing a pilot fall prevention program that could be implemented, sustained, and further improved. In keeping with previous work on translating research into practice [17], we drew on multiple theories, as well as previous experience, to plan program development and implementation. We built on organizational theory [18], previous efforts to set strategic priorities within the VA [19], diffusion of innovations theory [20], and (for implementation) principles of continuous quality improvement [21]. We discuss each of these elements in the paragraphs that follow.

Organizational theory helped us conceptualize the initial phase of program development. We drew upon a typology of organizational responses described in Oliver's "Strategic Responses to Institutional Processes" [18]. Oliver theorized that organizations respond in five typical ways to pressures to change business as usual: 1) acquiescence, 2) compromise, 3) avoidance, 4) manipulation and 5) defiance. She then laid out ten hypotheses as to what influences an organization's response to external pressure. Here, we focus on six of these hypotheses. Organizations are more likely to comply with external pressures if these pressures are viewed as 1) socially legitimate, 2) economically efficient, 3) not in conflict with other external demands on the organization, 4) consistent with institutional norms, 5) not constraining the organization's ability to act, and 6) voluntarily diffusing into the organization [18].

Oliver's framework and previous efforts at priority-setting in the VA system [19] suggested that our first development step should be to sound out leaders within our organization to verify that they valued implementing a fall screening and prevention program. Since organizational leaders represent organizations, their support is necessary (albeit not sufficient) for successful implementation. Second, whatever we proposed to leaders could not violate Oliver's hypotheses regarding which organizational changes are most likely to be acceptable. In our local context, this meant that we could not ask for new staff (which would violate the notion of economic efficiency), and we would need to spread the program's workload across all involved departments to avoid adversely affecting any one department.

The first step of the program development plan involved holding an initial meeting with VAGLAHS leadership, including the Chief of Staff (i.e., medical director). The goal of this first meeting was to ascertain whether managers within VAGLAHS agreed on a) the importance of fall prevention b) the strategies for preventing falls among community-dwelling veterans, and c) a broad outline of how these strategies might be accomplished. The first author's role in this process was to present evidence from the research literature about strategies for fall prevention, as well as the data on VA national and local performance in fall prevention, discussed earlier.

In our original program development plan, the next step was to hold focus groups of patients and employees who would likely be involved with the program. We did not hold these focus groups for two reasons. First, we did not hold focus groups of employees because we received feedback that employees might not be comfortable speaking candidly in the presence of other employees in the same organization. In addition, since the lead author had already conducted one-on-one interviews with multiple different employees prior to program development, re-interviewing employees seemed likely to have less incremental benefit than we originally had envisioned. Regarding patient focus groups, our status as a research study meant the need to obtain Institutional Review Board approval of focus group content and would have lengthened the program development process by three to six months. Since the program development process moved faster than anticipated, we decided not to pursue patient focus groups. Instead, we decided to use patient and stakeholder interviews to evaluate the first cycle of program development (discussed later).

The third step of the program development process was to constitute a workgroup of representatives from different disciplines involved in fall prevention to create a concrete plan. We aimed to identify workgroup members who represented the interests of various stakeholders, could provide insight regarding institutional norms and resources, and comprised a mix of content experts in fall prevention and clinicians and practitioners of quality improvement.

The fourth step was to hold a second leadership meeting to present the workgroup's suggested plan and receive feedback. Compared to the first meeting, this second meeting would be more focused on discussing specific options for the content of the fall prevention program, based on the findings of the workgroup.

Oliver's framework helped in choosing a program development and implementation process but did not directly address characteristics of a program itself that would make it more likely to be implemented successfully, issues that would matter to the workgroup. We used a systematic review of diffusion of innovations by Greenhalgh [20] to identify the following program attributes as being key for successful adoption:

• Simplicity--simpler innovations are more likely to be adopted than complex innovations

• Trialability--innovations that can be tested and experimented with are more likely to be adopted

• Observability--If adopters can see the benefits of an innovation, the innovation will be more easily adopted

• Reinvention--innovations that can be modified or refined to suit adopters' needs are more likely to be adopted

• Risk - innovations that are less risky are more likely to be adopted

In conformance with the principle of simplicity, we decided that any research on the program should not interfere with program delivery. In keeping with the principles of trialability, reinvention, observability, and risk minimization, we envisioned program implementation as being consistent with continuous quality improvement methodology, specifically including several concepts described by Locock:

• "Continuous incremental improvement of current processes--repeated testing and evaluation of small scale changes.

• Responsibility for quality in the hands of frontline staff.

• Collective team responsibility.

• Detailed meticulous measurement." [21]

This continuous quality improvement process would take place on an ongoing basis as the program was implemented.

## Results

The first leadership meeting (in February 2008) began with a ten-minute presentation of national data (Figure 1) and facility-level data. Seventeen people attended. At the meeting, multiple participants indicated that any new program should not "overwhelm the system" given a variety of competing clinical and quality improvement demands. The meeting closed with the Chief of Staff chartering a collaborative workgroup to develop a pilot outpatient fall prevention program.

The workgroup first met in April 2008, and consisted of at least one representative nominated from each of five departments: primary care, nursing, geriatrics, physical medicine & rehabilitation, and performance improvement. The first author, a researcher and clinician, served as a content expert regarding the evidence base for fall prevention. The workgroup met monthly and was attended by five to seven people at any given meeting.

**Figure 2:**
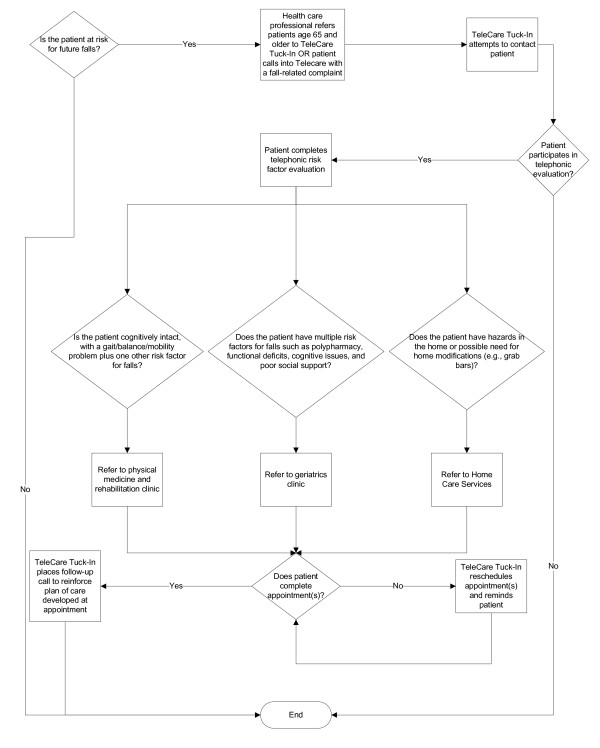
**Preliminary flow diagram of care model**. Relevant terms: *Telecare*: Telephonic advice line accepting incoming calls from patients with medical concerns. *Telecare Tuck-In*: Service that places outgoing calls to patients (typically at the request of a health care professional, but modified in this instance to follow up on relevant incoming calls by patients). *Home care services*: Arranges services provided in a patient's home, including any or all of the following services: home physical therapy, home occupational therapy, home safety evaluation, visiting nurse.

The first workgroup session involved brainstorming. Subsequently, a confidential ballot was sent by e-mail to workgroup participants to rank ideas from the brainstorming session, as well as some additional ideas that had come up in the interim (see additional file 1). The second workgroup meeting involved discussing the feasibility of the eight candidate ideas that received two or more votes on the ballot. Convergence on a lead idea for the fall prevention program occurred during the June 2008 workgroup meeting, a joint convening of the workgroup and representatives from Telecare, a telephonic nurse advice line that also offers outgoing calls to patients at a provider's request. Subsequent workgroup meetings led to the care model in Figure 2. The fall prevention program provides a central role for the Telecare Tuck-In Program (described below).

The second leadership meeting, held in October of 2008, involved vetting the care model and soliciting additional ideas; twenty-three people attended. The Telecare program screened its first patient in October of 2008 and continues to operate at this time.

### Description of the fall prevention program

The VA Desert Pacific Healthcare Network uses Telecare to field incoming symptom-related calls from patients. Telecare is staffed by nineteen registered nurses and eight clerks, and handles 12,000 to 13,000 calls per month from throughout the Network. Telecare Tuck-In is a component of Telecare; Tuck-In serves VAGLAHS only. In contrast to Telecare's main function of triaging incoming calls, the Tuck-In program places outgoing calls to patients at a healthcare professional's request. These calls might be to check on the patient's well-being or assess a patient's adherence to medications. Providers may only refer non-urgent cases to the Tuck-In program; if a patient is found to require urgent attention, the Tuck-In nurse triages the patient to an Emergency Department or urgent care clinic.

For the fall prevention program, health care professionals from anywhere in VAGLAHS can place a referral to Telecare Tuck-In program to evaluate a patient's risk factors for falls. The Telecare Tuck-In registered nurse then calls the patient and reads him/her (or his/her caregiver) a standard set of scripted questions to assess risk factors for falls, such as whether the individual has impaired vision, gait or balance problems, or need for home modifications (see Additional File 2). The nurse, using a pre-determined algorithm based on the care model in Figure 2, then requests an appointment for the patient for the physical medicine & rehabilitation falls clinic, geriatrics clinic, and/or home care services. The script and algorithm were developed with the idea of conducting a simplified multifactorial assessment for fall risk factors [6] and tailoring interventions to the patient's needs. Due a need for brevity and the telephonic basis of the assessment, the workgroup decided that not all critical risk factors could be fully assessed.

One month after the initial contact, the Telecare Tuck-In nurse then places a second phone call to the patient to assess the patient's adherence to appointments. Telecare's medical director agreed to support the Telecare Tuck-In nurse in cases where medical authorization is needed for appointments. However, to maintain continuity of care, primary care providers are to be electronically alerted to any notes or referrals completed by the nurse. For the pilot phase of the program, Telecare has also been screening incoming calls from patients who might have a fall-related concern and routing them to the Telecare Tuck-In nurse for further evaluation.

The Telecare fall prevention program's main advantage is its ability to coordinate existing resources for fall prevention at VAGLAHS. Because Telecare conducts business by phone, it can accept referrals from anywhere in VAGLAHS, even geographically remote areas. Another potential advantage of the Telecare fall prevention program is the opportunity to unburden primary care providers of additional responsibilities by helping assess patients' needs and arranging the appropriate services.

Areas currently being explored include determining the best combination of referral sources to the program (e.g., Telecare itself, primary care, patient self-referral, urgent care, or the Emergency Department) as well as which individuals will most commonly make referrals (e.g., social workers, nurses, physicians, or patients self-referring). Another area that requires additional exploration is the best way to routinely track completion of recommended activities (e.g., home safety evaluation) for patients who participate in the program. In addition, a new phase of the program, encouraged by workgroup participants, is starting (see additional file 3).

### Program evaluation

The fall prevention workgroup, which has continued beyond its initial 6-month charter, serves as a forum for ongoing program evaluation. Participation is now voluntary, and the group meets via conference call to facilitate participation. Workgroup meetings are used to troubleshoot emerging issues (e.g., timely completion of a fall prevention referral to Telecare), as well as to discuss new ideas. Five to nine participants typically join each call.

In addition to the ongoing evaluation that occurs as part of the Telecare fall prevention program itself, a research evaluation of the Telecare fall prevention program is planned. This research evaluation will involve reviewing a log to see how many individuals were screened for the program, as well as performing interviews with patients who participated in the program. The evaluation will also interview stakeholders who help run the program, refer patients to it, or are referred patients from the program. We will assess interviewees' satisfaction with the program as well as solicit ideas for improvement. The evaluation will also review patients' medical records to assess how well the pilot program is being implemented, as well as the overall quality of care patients received for fall prevention. Medical record review will use quality indicators for care of vulnerable older adults with falls and mobility disorders [22], for which benchmark data from non-VA sites are available [23]. Given the incremental approach we have taken, we do not expect the evaluation of the first iteration of the Telecare fall prevention program to demonstrate uniformly positive findings, but do think it will yield more concrete areas for improvement efforts.

## Discussion

Multifactorial fall programs are complex to implement in the outpatient setting because they require cooperation between multiple healthcare professionals as well as changes in patients' behavior. A recent organizational survey of managers in the United Kingdom attests to the difficulties in implementing multifactorial fall prevention programs in routine care, even when there is a national mandate to do so [24]. With respect to our own program, we are concerned by recent data suggesting that programs that directly mitigate patients' risk factors for falls may be more successful than those that depend on patients to complete referrals to other providers [25]. Indeed, a recent process evaluation of a fall prevention program with negative results suggests that patients' failure to follow up with their general practitioner was an important reason that the study was not implemented as planned [26]. As a consequence, a significant part of our program evaluation will focus on whether our patients are completing referrals made for them.

We have some early observations about program development. First, our program development and implementation process has been non-linear. For the first six months, the workgroup's intensity of effort was quite high, and then settled into a maintenance phase after implementation of the program had begun. Second, program development was a hybrid of "bottom-up" and "top-down" approaches [27]. Although the workgroup functioned autonomously once it was chartered, we benefited crucially from leadership support for creation of the workgroup. For example, we explicitly sought and received support from our local facility's Chief of Staff for our efforts. In addition, decisions by VA national leadership to measure and internally report facility-level quality of care for falls provided us with evidence to convince our local facility's leadership that care should be improved.

Third, the program development process unfolded over the good part of a year, and we are still early in the process of implementation. Although slow, this pace is likely to improve sustainability, since participants carry out their program-related duties as part of their usual workload. Fourth, we lack easily obtainable metrics to determine whether care is improving. This contrasts with inpatient fall prevention programs, where data on fall and injury rates are often captured as part of adverse event reporting systems, and can be tracked quarterly to measure response to incremental changes made to improve care. In the outpatient setting, it can be difficult to capture detailed data on falls without turning a quality improvement program into a research study, since most falls in community-dwellers occur outside the four walls of the healthcare system and may not be reported by the patient to a health care provider until months after the fall occurred. Instead, we are relying on continuous quality improvement strategies on a real-time basis to monitor intermediate steps in the process of care, such as completion rates for home safety evaluations that are ordered. In the future, it may be possible to shorten improvement cycles with real-time measures of quality of care for falls using electronic health record data.

Last, we anticipate new questions with regard to diffusion and dissemination of the program should our initial evaluation of the program prove positive. Even though our program development process uses existing staff to implement the program, we still benefit from highly motivated individuals who have willingly taken on new duties to improve the quality of care. Although participants in the workgroup may have participated because of a supervisor's request, the degree of participants' involvement has been determined by their own level of interest in and commitment to fall prevention. Extending quality improvement activities beyond the immediate environments that can be affected by workgroup participants to other areas of our system could prove challenging. When we have our initial evaluation results, we plan to re-approach our facility's clinical leadership and discuss possible next steps for diffusion and dissemination.

## Conclusion

Our program development process is proving sustainable thus far. Since its inception, the multidisciplinary workgroup has continued to meet monthly. The goal of the program development process was to create an initial effort that was sustainable enough to demonstrate a small success to our organization, generating goodwill and the opportunity to improve the program over time, in keeping with continuous quality improvement principles [28]. By these criteria, we believe the initial phase of the development process has been a success.

## Competing interests

The authors declare that they have no competing interests.

## Authors' contributions

DAG, EMY, DS, and PGS developed the conceptual framework for the program development and implementation process. EMY and DG analyzed the External Peer Review Program data. DAG drafted the manuscript. EMY, DS, and PGS provided comments for critical revision of the manuscript. All authors read and approved the final manuscript.

## Pre-publication history

The pre-publication history for this paper can be accessed here:

http://www.biomedcentral.com/1472-6963/9/206/prepub

## Supplementary Material

Additional file 1 Instructions and ballot sent to workgroup participants. Click here for file

Additional file 2 Patient script used by Telecare Tuck-In nurse. Click here for file

Additional file 3 Description of new fall prevention program elements, including nurse clinical reminder to assess fall risk in patients age ≥ 75 and electronic health record menu options for fall prevention. Click here for file
