# Supplementary material for: Design of a continuous quality improvement program to prevent falls among community-dwelling older adults in an integrated healthcare system
Source: BMC Health Serv Res. 2009 Nov 16;9:206. doi: 10.1186/1472-6963-9-206 (PMC2779811; doi:10.1186/1472-6963-9-206)
Supplement: Additional file 1 — Instructions and ballot sent to workgroup participants. [file 1472-6963-9-206-S1.DOC]

**Additional file 1.** Instructions and ballot sent to workgroup participants.

Please review the list of ideas generated by the fall workgroup on 4/25/08 as well as some additional ideas that emerged since then, and choose your **top five ideas** to further explore. Mark those ideas with an “X” in the second column and return your completed ballot to David Ganz via e-mail ([dganz@mednet.ucla.edu](mailto:dganz@mednet.ucla.edu)).

While reviewing the ideas, keep in mind that the following programs have been shown to reduce falls: 1) exercise programs, and 2) multifactorial programs that include a medical and home safety evaluation with appropriate interventions driven by what’s found on the evaluation. A comprehensive fall risk reduction program should thus cover the three domains of physical activity, medical evaluation and home safety. In addition, patient education and activation is necessary to support all of these activities.

**Ideas from first workgroup meeting on 4/25/08:**

Interventions targeted at patients:

| - Taking advantage of Patient Education Resource Center resources |  |
| --- | --- |
| - Working with families of patients |  |
| - Providing group information on buses and vans to/from VA facilities using video, audio, or personal conveyance of information |  |
| - Checking in with patients to show caring after an outpatient visit |  |
| - Inserts about fall risk into mail order pharmacy packages that are mailed out to patients |  |
| - Pre-visit questionnaires filled out by patients |  |
| - Using birthday postcards mailed out by an automated system to activate patients about fall prevention |  |
| - Using tele-reminder system to leave phone messages with patients |  |
| - Using existing telehealth systems (e.g., Health Buddy) as a vehicle for inquiring about fall risk and/or activating patients on issues of fall risk |  |

Interventions targeted at providers:

| - Clinical reminders (general consensus against) |  |
| --- | --- |
| - Training of wheelchair escorts to educate patients about wheelchair falls |  |
| - Educating nurses who answer the nurse triage phone line about how to handle fall-related inquiries |  |
| - Educating ambulatory case managers to be aware of fall risk when screening patients’ charts |  |

Interventions targeted at the level of the organization:

| - Linking with Home-Based Primary Care/home health to do home safety |  |
| --- | --- |
| - Linking with the Managing Overweight and/or Obesity for Veterans Everywhere (MOVE!) initiative |  |
| - Linking with inpatient “difficult-to-discharge patient” initiative |  |
| - Linking with Telecare “tuck-in” program (calling patients in the evening to check in) |  |
| - Taking advantage of the inpatient to outpatient transition – dedicated home physical therapy/occupational therapy evaluation at home prior to patient’s discharge |  |
| - Developing links to community (i.e., outside the VA) programs |  |
| - Using teleconsultation to support the needs of community-based outpatient clinic providers when patients have limited access to specialty resources |  |
| - Building an automated fall risk prediction system using administrative and/or electronic health record data to target high risk patients |  |
| - Using fall injuries as an outcome measure to monitor progress of interventions to prevent falls |  |
| - Linking osteoporosis and fall risk screening |  |
| - Linking with prosthetics department |  |

Other ideas that have come up since the workgroup meeting:

| - Targeting patients who present to the Emergency Department with a fall for possible intervention (e.g., home safety evaluation) |  |
| --- | --- |
| - Computerized provider order entry to warn providers about high-risk medications |  |
| - Expedited referral to physical therapy |  |
| - Maximizing patients’ co-existing Medicare Part B benefits (where applicable) to help patients get appropriate fall-related evaluation (e.g., outpatient physical therapy, home health) |  |
| - Developing local community resource lists for each clinic that can be given to the patient in the form of a handout |  |
