# Supplementary material for: Design of a continuous quality improvement program to prevent falls among community-dwelling older adults in an integrated healthcare system
Source: BMC Health Serv Res. 2009 Nov 16;9:206. doi: 10.1186/1472-6963-9-206 (PMC2779811; doi:10.1186/1472-6963-9-206)
Supplement: Additional file 2 — Patient script used by Telecare Tuck-In nurse. [file 1472-6963-9-206-S2.DOC]

**Additional file 2**. Patient script used by Telecare Tuck-In nurse. A similar script exists for caregivers if the patient is not able to answer questions.

Relevant abbreviations: CPRS, Computerized Patient Record System.

**1a. In the past year, have you fallen?** **Yes** **No**

**1b.** (If yes to 1a) **With the fall, did you hurt yourself or need to see a doctor because of a fall?** **Yes** **No**

**2a.  Do you have problems with walking or balance?** **Yes** **No**

**2b.** (If yes to 2a) **Would you like a medical expert to check your walking and balance to see if you can improve them?** **Yes** **No**

**3a.  Do you use a walking aid, such as a cane or walker?** **Yes** **No**

**3b.** (If yes to 3a) **Has a doctor, nurse or therapist showed you how to use your cane or walker?** **Yes** **No**

**3c.** (If no to 3a) **Has a doctor, nurse or therapist recommended you use a cane or walker?** **Yes** **No**

4.  (Review medication list in CPRS. Assess for use of benzodiazepines, such as temazepam, lorazepam, diazepam. If a patient is listed as taking a benzodiazepine, ask the following:)

**4a. Are you still taking a medicine called [name of medicine]?** **Yes** **No**

**4b.** (If yes to 4a) **Would you be willing to meet with a doctor to talk about whether you still need to be on this medicine?** **Yes** **No**

**5a.  Do you feel dizzy, woozy, or lightheaded when you sit up or stand up?** **Yes** **No**

**5b.** (If yes to 5a) **Has this happened more than 4 times in the past month?**

**Yes** **No**

**5c.** (If yes to 5b) **Would you be willing to see a doctor for this problem?**

**Yes** **No**

6.  (Look up whether the patient has had an eye exam within the past year in CPRS. If not, ask the following:)

**6a. Do you have problems with your vision?** **Yes** **No**

**6b.** (If yes to 6a) **When was your last appointment with an eye doctor?**

**Record date here:       Today’s date:**

(If no appointment at the VA or an outside provider in the past year, ask the following:)

**6c. Would you like to see an eye doctor to check your vision?** **Yes** **No**

**7a.** **Do you need help to use the bathtub, shower, or toilet?** **Yes** **No**

**7b.** (If yes to 7a) **Would you like someone to teach you ways to make using the bathtub, shower, or toilet easier?** **Yes** **No**

**7c.** (If yes to 7a) **Do you have grab bars in your bathroom?** **Yes** **No**

**7d.** (If no to 7c) **Would you be interested in having someone visit your home to see whether grab bars should be put in?** **Yes** **No**

**8a.** **Have you been having memory problems?** **Yes** **No**

**8b.** (If yes to 8a) **Would you like to see a doctor to check your memory?**

**Yes** **No**

**9. Are there any other problems that you’re having that you’d like to discuss with me?**

**10. Would you like more information mailed to you about how to prevent falls?** **Yes** **No**
