# Supplementary material for: Design of a continuous quality improvement program to prevent falls among community-dwelling older adults in an integrated healthcare system
Source: BMC Health Serv Res. 2009 Nov 16;9:206. doi: 10.1186/1472-6963-9-206 (PMC2779811; doi:10.1186/1472-6963-9-206)
Supplement: Additional file 3 — Description of new fall prevention program elements, including nurse clinical reminder to assess fall risk in patients age ≥ 75 and electronic health record menu options for fall prevention. [file 1472-6963-9-206-S3.DOC]

**Additional File 3.**

A second phase of the fall prevention project launched in March 2009. Nurses in several community-based outpatient clinics are screening for fall risk (see screenshot of nurse clinical reminder to assess fall risk in patients age ≥75), and pass on the results of positive screens to providers. These providers received an in-service on fall prevention from a geriatrician, who is a member of the workgroup, the clinical champion for this phase of the project, and a full-time primary care provider in one of the affected clinics. The in-service focused on how to integrate fall evaluation into providers’ clinical routine and builds on previous work in this area.[[1]](#footnote-2) In addition, a specially designed list of referrals typically made for patients at fall risk (including the Telecare fall prevention program) is available to providers in their electronic health record to simplify the task of remembering which clinical actions to take in response to a positive screen (see screenshot of electronic health record menu options for fall prevention below).


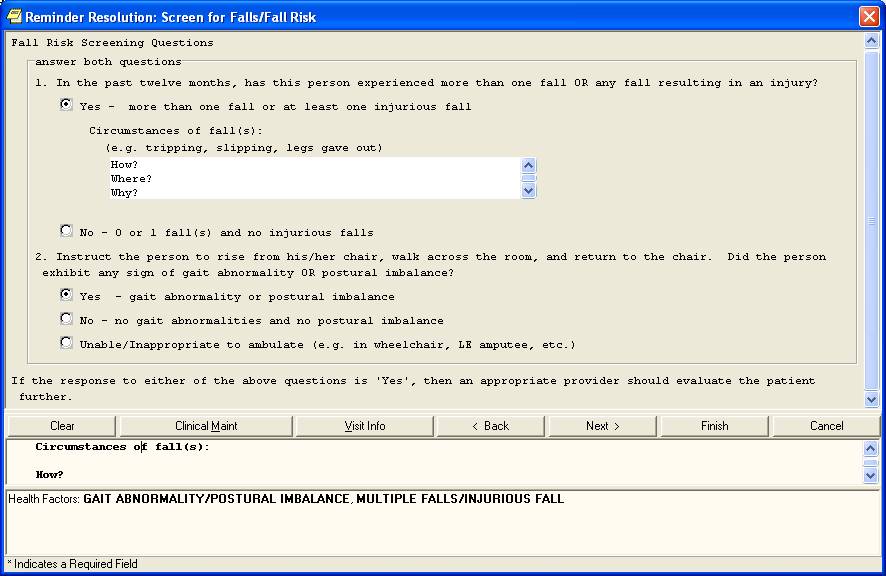


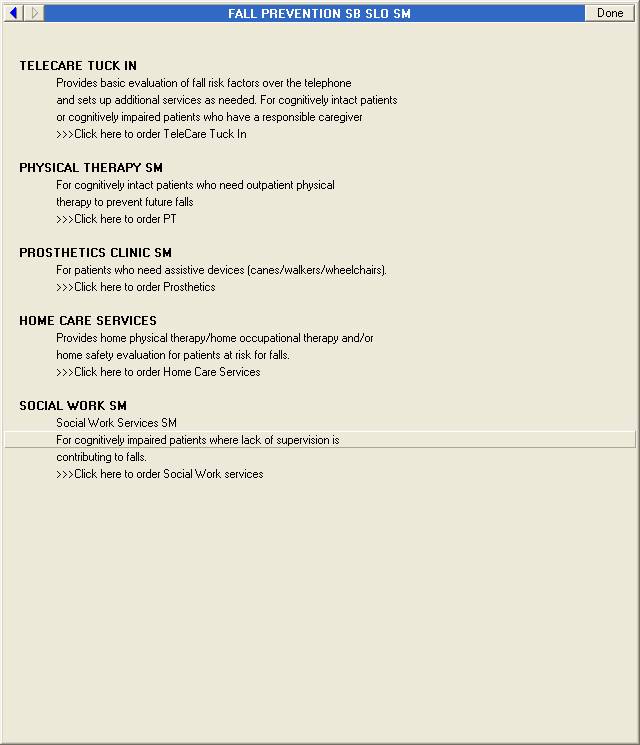


1. Reuben DB, Roth C, Kamberg C, Wenger NS: **Restructuring primary care practices to manage geriatric syndromes: the ACOVE-2 intervention**. *J Am Geriatr Soc* 2003, **51**(12):1787-1793. [↑](#footnote-ref-2)
